# Acknowledgement to Reviewers of *Foods* in 2014

**DOI:** 10.3390/foods4010001

**Published:** 2015-01-08

**Authors:** Foods Editorial Office

**Affiliations:** MDPI AG, Klybeckstrasse 64, CH-4057 Basel, Switzerland

The editors of *Foods* would like to express their sincere gratitude to the following reviewers for assessing manuscripts in 2014:

Ansorena, Diana
Arvanitoyannis, Ioannis
Aydin, Senay
Babic, Jurislav
Baiano, A.
Barding, Gregory
Barthelmebs, Lise
Bauer, Friedrich
Beermann, Christopher
Belobrajdic, Damien P
Bermúdez-Aguirre, Daniela
Boileau, Amy
Botsoglou, Evropi
Calvete, Juan J.
Capita, Rosa
Casal, Susana
Casanueva, Felipe F
Chohan, Magali
Christensen, Larry
Chruszcz, Maksymilian
Ciardiello, Maria Antonietta
Contreras-Padilla, Margarita
Copeland, Les
Cruz, Adriano G.
Cunsolo, Vincenzo
Curiel, J. A.
da Cruz, Adriano Gomes
Dais, Photis
Daniel, James R.
Delwiche, Stephen R.
Denicola, Ana
Diaz-Rubio, M. Elena
Dykes, Gary
Elez-Martinez, Pedro
Engel, Karl-Heinz
Erdoğdu, Ferruh
Farid, Mohammed M.
Fessas, Dimitrios Fessas
Fotakis, Charalambos
Fraqueza, Maria João
Fusco, Vincenzina
Geveke, David
Giaouris, Efstathios
Gómez-Gallego, Carlos
Gottschalk, Klaus
Haesaert, Geert
Heller, Martin C.
Hill, Andrew
Holck, Askild Lorentz
Holick, Michael F.
Holley, Richard A.
Hospital, Xavier F.
Jenkins, Rowena
Jensen-Jarolim, Erika
Jooken, Etienne
Khalid, Nauman
Kiely, Mairead
Kitts, David D.
Klein, Gunter
Kneil, Kali
Kocatepe, Demet
Kuhn, David
Lebot, Vincent
Lee, C. B. Alvin
Lobo Castañón, Maria Jesus
Maddocks, Sarah E.
Mandala, Ioanna
Marsellés Fontanet, Robert
Martinez, Maribel
Martínez-Cuesta, M. Carmen
Maulvault, Ana Luisa
Meier, Toni
Mine, Yoshinori
Monaci, Linda
Moughan, Paul J.
Moulisova, Vladimira
Moutsatsou, P.
Nemeghaire, Stéphanie
Odabas, Serhat
Ozsisli, Bahri
Palou, Andreu
Panagou, Efstathios Z.
Paulsen, P.
Pelchat, Marcia Levin
Perussello, Camila Augusto
Petrakis, Panos. V.
Pouliot, Yves
Purlis, Emmanuel
Rajagopal, S. N.
Rakwal, Randeep
Raphaelides, S. N.
Reuter, Andreas
Routray, Winny
Salgo, Andras
Salmerón, Ivan
Sato, Kenji
Schaafsma, Gertjan
Shah, Romina
Simitzis, Panayiotis
Singh, Amit
Siwela, Muthulisi
Smith, Scott
Speight, Natasha
Thomas, Raymond H.
Thompson, Harvey
Tormod, Mørk
Torres, J. Antonio
Trystram, Gilles
Vélez-Ruiz, Jorge F.
Viswanathan, Subramanian
Wang, Chung-Yi
Wang, Rong
Weyermann, Maria
Wilkinson, Brian
Xiao, Hui
Yang, Hongshun
Yaylayan, Varoujan Antranik
Young, Howard
Zimmermann, Benno F.

